# Gene polymorphism of cytochrome P450 significantly affects lung cancer susceptibility

**DOI:** 10.1002/cam4.2367

**Published:** 2019-07-01

**Authors:** Meng Li, Anqi Li, Ruiqing He, Wenhui Dang, Xinyu Liu, Tian Yang, Puyu Shi, Xiang Bu, Dan Gao, Ning Zhang, Shuli Du, Tianbo Jin, Mingwei Chen

**Affiliations:** ^1^ The Department of Respiratory and Critical Care Medicine First Affiliated Hospital of Xi'an Jiaotong University Xi'an Shaanxi China; ^2^ Shaanxi Provincial Research Center for the Project of Prevention and Treatment of Respiratory Diseases Xi'an Shaanxi China; ^3^ The Department of Clinical Laboratory First Affiliated Hospital of Xi'an Jiaotong University Xi'an Shaanxi China; ^4^ Ministry of Education Key Laboratory of Resource Biology and Biotechnology in Western China Northwest University Xi'an Shaanxi China

**Keywords:** cytochrome P450 (CYP450), genetic polymorphism, lung cancer, susceptibility

## Abstract

**Background:**

Cytochrome P450 (CYPs) are heme proteins involved in the metabolism of a variety of endogenous and exogenous substances and play an important role in the carcinogenesis mechanisms of environmental and hereditary factors. The objective of this study was to investigate how polymorphisms of CYPs correlate with lung cancer (LC) susceptibility.

**Methods:**

Six single nucleotide polymorphisms (SNPs) were genotyped in this study. The chi‐square test and unconditional logistic regression model were used to evaluate the correlation between SNPs and LC susceptibility. The expressions and survival data of genes in patients with LC were mined using Oncomine and Kaplan‐Meier Plotter database.

**Results:**

Four SNPs were found to be significantly associated with the risk of LC development (*P *< 0.05). The most significant correlation was that the A allele and AA genotype of *CYP2D6* rs1065852 were associated with increased risk of LC development (adjusted odds ratio [OR] = 1.35, 95% confidence interval [95%CI] = 1.13‐1.60, *P *= 9.04e‐4; OR = 1.83, 95%CI = 1.29‐2.59, *P *= 0.001 respectively). Similar association of this variant was also found in the subgroups of male patients, cases in III‐IV stages, positive lymph node, squamous cell carcinomas and adenocarcinomas. Whereas rs1065852 was considered as protective factor in females (adjusted OR = 0.33, 95% CI = 0.16‐0.70, *P *= 0.004). In stratified analyses, the association of *CYP24A1* rs2762934, *CYP24A1* rs6068816, *CYP20A1* rs2043449 polymorphism with LC risk appeared stronger in some subgroups. *CYP2D6*, *CYP24A1* and *CYP20A*1 are overexpressed in some pathological types of LC (*P *< 0.05), and high levels of *CYP2D6* and *CYP20A1* indicate poor and good prognosis of LC, respectively.

**Conclusion:**

This study revealed that rs1065852, rs2043449, rs2762s934, and rs6068816 of CYPs were associated with LC susceptibility in the Northwestern Chinese Han population; *CYP2D6* and *CYP20A*1 were overexpressed and correlated with prognosis of LC.

## 1 INTRODUCTION

Cancer constitutes a burden all over the world.^1^ It is estimated that nearly half of the new cases and more than half of the cancer deaths in the world will occur in Asia in 2018.^2^ Lung cancer (LC) is the most common cancer which accounts for 11.6% of cancer cases, and is the leading cause of male patient deaths which account for 18.4% of cancer death, especially in East Asia and Polynesia.^2^, ^3^ Cigarette use remains the primary causal agent of LC,^4^ however, other susceptibility factors such as ionizing radiation, air pollution, and exposure to occupational and environmental carcinogens, such as radon and formaldehyde could also increase the incidence of LC.

Nowadays, an increasing number of studies show a strong link between genetic factors and carcinogenesis.^5^, ^6^, ^7^ Genome‐wide association studies (GWAS) have been identified several susceptibility gene locus of cancer in European people, including CHRNA3/5, CHRNB4, BRCA2, CHEK2, TERT, but only a small part of LC genetic capacity can be proved by these gene loci, and most have not been systematically verified in Asian populations.^8^, ^9^, ^10^ Since it is the ethnic group with the largest population in East Asia, it is crucial to explore the relationship between genetic polymorphisms and susceptibility of LC in the Chinese Han population.

The cytochrome P450 superfamily (CYPs), located primarily in the liver, small intestine and kidney,^11^ is a large superfamily of integral membrane conserved proteins present in animals, plants, and microorganisms,^12^ which play a crucial role in the metabolism and activation of carcinogens.^13^ All these active carcinogens can combine with DNA and form DNA adducts which are capable of inducing mutations and initiating tumorigenesis. Genetic polymorphisms of CYPs have been reported to be associated with various diseases and adverse drug reactions among different populations by affecting the enzyme catalytic activity.^14^, ^15^ Kiyohara C has found that the CYPs genetic polymorphism is related to the susceptibility of colorectal cancer.^16^ Maurya et al reported that polymorphisms of drug metabolizing CYPs showed modest associations with head and neck squamous cell carcinoma risk.^17^ Genetic polymorphisms have been reported for CYPs involved in the metabolic activation of polycyclic aromatic hydrocarbons (PAHs) and tobacco‐specific nitrosamines,^18^, ^19^ both of which are wide spreading environmental procarcinogens that induce LC and skin carcinoma.^20^, ^21^, ^22^ However, Kiyohara C et al have found no significant association between the genetic polymorphism of enzymes involved in xenobiotic metabolism and the risk of LC.^23^

To sum up, the correlation of CYPs polymorphisms and LC risk is contradictory and inconclusive due to the diversity of ethnicity and sample size in study groups. In order to validate the association between genetic polymorphisms of CYPs and susceptibility to LC in Northwest Chinese Han population, we adopted a case‐control study and selected six SNPs associated with cancer from the target enzyme system to genotype and evaluate the impact of CYPs genetic polymorphisms on the risk of LC development in general and different subgroups concerning gender, tumor stages, lymph node status, and pathologies. The gene expression and relationship between the expression level and prognosis of LC were further analyzed using Oncomine and Kaplan‐Meier plotter database.

## 2 MATERIALS AND METHODS

### 2.1 Subject and ethics statement

Five hundred and ten Pathologically confirmed LC patients hospitalized in the First Affiliated Hospital of Xi'an Jiaotong University, Shaanxi, China, were included in this study (both SCLC and NSCLC were included). Tumor stages and pathological classifications were based on the 8th edition of TNM staging system published by the Union for International Cancer Control and pathological results respectively.^24^ Relevant information was extracted from medical files. Patients with other tumors and communication problems were excluded. Five hundred and four healthy subjects were recruited into the cancer‐free control group in the same hospital at the same time, none of them had any history of cancers, severe endocrine and autoimmune diseases. It was made sure that there was no genetic relationship between the cases and the control subjects, the purpose of which was to minimize the environmental, hereditary and therapeutic factors affecting genetic susceptibility to LC. This study strictly complies with the Helsinki declaration of the World Medical Association. The cases and the subjects of the control group provided consent and the research was approved by the Ethics Committee of The First Affiliated Hospital of Xi'an Jiaotong University.

### 2.2 SNPs selection and primer design

Six SNPs from three genes of CYPs associated with LC were selected for analysis in this study based on 1000 genome projects. Each of them met the criteria of the minimum allele frequency (MAF), more than 5%, in the HapMap of the Chinese Han Beijing population. All primers were designed using ASSAY DESIGN SUITE V2.0. (http://agenacx.com/online-tools, Table 1).

**Table 1:** Primers of candidate SNPs

SNP ID	First‐PCR primer sequences	Second‐PCR primer sequences	UEP‐DIR	UEP sequences
rs2043449	ACGTTGGATGTGCCACCACCAGATTGATAC	ACGTTGGATGACAGGGTATCTTTTTGAGTG	Forward	GAAATATTTAATTTGTCTGTTTCAG
rs2762934	ACGTTGGATGGTTCCAGAAGCTGTACTGTC	ACGTTGGATGTGTAGAATGCCTTGGATCCC	Forward	CCCAGCACTCAGTCC
rs1570669	ACGTTGGATGAGACGAAGTTGAGGCTCACA	ACGTTGGATGGAATTCACGGCTATGGAGAC	Reverse	CCTCGACCTGCATTCAGTTTCA
rs6068816	ACGTTGGATGCTTCCAGAACGAACATTGTC	ACGTTGGATGCGACTGGAGTGACCATCATC	Forward	CCCTCCCATCATCCTCCCAAA
rs2296241	ACGTTGGATGAAATGTGTCTTTTGCGGTTG	ACGTTGGATGTCTTCAACGTGGCCTCTTTC	Forward	TCATCTATTCTGCCCATAAAATC
rs1065852	ACGTTGGATGTGGAAGTCCACATGCAGCAG	ACGTTGGATGTGCTCCTGGTGGACCTGATG	Reverse	CTCCCACGCTGGGCTGCACGCTAC

Abbreviations: SNP, single nucleotide polymorphism; UEP‐DIR, unextension primer sequence direction.

### 2.3 SNPs genotyping and haplotype analysis

Genomic DNA was extracted from peripheral blood using GoldMag‐Mini Whole Blood Genomic DNA Purification Kits (GoldMag Co. Ltd., Xi'an City, China), and quantified with a spectrophotometer (NanoDrop 2000; Thermo Fisher Scientific, Waltham, MA, United States). To have sufficient DNA for further reactions, polymerase chain reaction (PCR) was applied to each sample. Then SAP purification was performed to remove the remaining dNTP and amplified primers in PCR products. Using a MassARRAY Nanodispenser (Agena Bioscience, San Diego, CA), standardized genotyping reactions were dispensed onto a 384‐well spectroCHIP. The repeated control samples were set in every genotyping plate and the concordance was more than 99%. The genotyping of these SNPs was carried out on the MassARRAY iPLEX (Agena Bioscience, San Diego, CA) platform using the allele‐specific matrix‐assisted laser desorption ionization‐time of flight mass spectrometry (MALDI‐TOF‐MS). Genotyping results were outputted by Agena Bioscience TYPER 4.0. Haploview software package (version 4.2) was used to analyze the linkage disequilibrium (LD), haplotype construction, genetic association at polymorphism loci and haplotype blocks were defined according to the criteria laid out by Gabriel and others.^25^

### 2.4 Statistical analysis

Data were analyzed by using SPSS 18.0 statistical software (SPSS Inc, Chicago, IL) and Microsoft Excel (Microsoft Corp., Redmond, WA). All continuous data are expressed as means ± standard deviations (SDs). Pearson's χ^2^ test and *t* test were used to compare the distribution of categorical variables and continuous variables between the cases and controls respectively.

The lower frequency alleles were encoded as the minor allele. Frequencies of all SNPs in both case and control groups were evaluated for the test of Hardy–Weinberg Equilibrium (HWE). The three genetic models (dominant, recessive and additive) were applied using PLINK software (http://www.cog-genomics.org/plink2/) to assess the association of single SNPs with the risk of LC development. The odds ratios (ORs) and 95% confidence intervals (95% CIs) were calculated by using logistic regression analysis and were adjusted for age and gender. To explore the possibility that the effect of a genetic polymorphism in candidate genes may be biologically active only in some specific subgroups, we conducted stratified analyses investigating the effect of genotype within the gender, lymph node status, tumor stage and histological subtypes based on medical reports. Exploratory analyses examining the effect of genetic polymorphisms within the histological subtypes based on pathology reports were also conducted. Statistical significance was identified at *P* ≤ 0.05 (two‐side). Power and Sample Size (PS) Calculation software (http://biostat.mc.vanderbilt.edu/wiki/Main/PowerSampleSize) was used to calculate the power of the significant difference.^26^ We estimated the association of haplotype with the susceptibility to LC using PLINK software (http://www.cog-genomics.org/plink2/). The ORs and 95% CI were also calculated using unconditional logistic regression analyses adjusted for age and sex.

### 2.5 Gene expression and survival analysis

Expression and survival data of candidate genes in LC patients were mined using ONCOMINE (https://www.oncomine.org/resource/login.html) and Kaplan‐Meier Plotter (http://kmplot.com/analysis/) database. The Kaplan‐Meier method and Cox regression were performed to construct survival curves and estimate hazard ratios (HRs) to assess the relationship between risk genes expression and prognosis of LC.

## 3 RESULTS

### 3.1 Baseline characteristics

A total of 1014 participants were included in the study, 510 patients with LC in the case group (384 males and 126 females; average age: 58.08 ± 10.55 years) and 504 healthy subjects in the control group (381 males and 123 females; average age: 57.27 ± 10.85 years). Characteristics of patients in the case group and the subjects in the control group included in this study are listed in Table 2, There was no significant difference in distribution of gender and age between the two groups (*P* = 0.911; 0.227 respectively).

**Table 2:** Characteristics of cases and controls in the study

	Case (N = 510)		Control (N = 504)		*P‐*value	
	Case %		Count %			
Sex						
Male	384	75.3	381	75.6	0.911^a^	
Female	126	24.7	123	24.4		
Age (y)						
Mean ± SD	58.08 ± 10.55		57.27 ± 10.85		0.227^b^	
TNM stage						
I‐II	129	25.3				
III‐IV	248	48.6				
Miss	133	26.1				
Lymph node status						
Positive	193	37.8				
Negative	120	23.5				
Unknown	197	38.6				
Type of lung cancer						
SCC	169	33.1				
AC	161	31.6				
SCLC	97	19.0				
Others	22	4.3				
Miss	61	12.0				

Notes

*P *≤ 0.05 indicates statistical significance; *P* ^a^ values were calculated from two‐side chi‐square test; *P* ^b^ values were calculated by *t* tests; Miss indicates data loss.

Abbreviations: AC, adenocarcinoma; SCC, Squamous cell carcinoma; SCLC, small cell lung cancer; TNM, tumor‐lymph node‐metastasis.

### 3.2 Linkage between candidate gene polymorphisms and LC

Six SNPs of CYPs were identified. The success ratio was >99.40% for all SNPs. Primary information of the candidate SNPs is shown in Table 3. No significant deviation of genotype frequencies for CYPs from the HWE was found in both groups (Table 3). The A allele of rs1065852 in *CYP2D6* was associated with a 0.35 times increased risk of LC development in the allelic model analysis with power values of 0.937 (adjusted OR = 1.35, 95%CI = 1.13‐1.60, *P* = 9.04e‐4).

**Table 3:** Candidate SNPs tested in cancer‐related genes

SNP ID	Chr	Gene (S)	Position	Allele A/B	MAF Case Control		*P*‐_HWE_	Call rate (%)	OR (95%CI)	*P*‐adj^a^	Study power
rs2043449	2	CYP20A1	203,251,967	C/T	0.08	0.07	0.721	100%	1.25 (0.90‐1.74)	0.179	
rs2762934	20	CYP24A1	54,154,722	A/G	0.09	0.11	0.818	100%	0.82 (0.61‐1.09)	0.175	
rs1570669	20	CYP24A1	54,157,888	A/G	0.39	0.39	0.453	100%	1.01 (0.85‐1.21)	0.879	
rs6068816	20	CYP24A1	54,164,552	T/C	0.35	0.33	0.686	99.4%	1.11 (0.92‐1.33)	0.274	
rs2296241	20	CYP24A1	54,169,680	A/G	0.44	0.44	0.786	99.7%	0.98 (0.83‐1.17)	0.852	
rs1065852	22	CYP2D6	42,130,692	A/G	0.52	0.44	0.928	99.4%	**1.35 (1.13‐1.60)**	9.04e‐4^*^	**0.937**

Notes

*P*‐adj: were adjusted by age and sex; ^*^ *P *≤ 0.05 value indicates statistical significance.

Bold indicates the siginificant value *P* ≤ 0.05 and the value of study power>0.8

Abbreviation: CI, confidence interval; HWE, Hardy‐Weinberg Equilibrium; MAF, minor allele frequency; ORs, odds ratios; SNP, single nucleotide polymorphism.

### 3.3 Linkage between candidate SNPs and LC development in genetic models and Haplotype analysis

We further conducted logistic regression analysis tests to analyze model associations. For SNP rs1065852 in *CYP2D6*, the genotype frequency distributions were different between the case group and control group (*P* = 0.003, Table 4). We ascertained that the AA genotype of rs1065852 in *CYP2D6* has a risk effect of promoting LC development compared with other genotypes. Homozygous mutations (AA) of rs1065852 in CYP2D6 increased the LC susceptibility by 1.83 times with power values of 0.998 (adjusted OR = 1.83, 95% CI = 1.29‐2.59, *P* = 0.001) compared with individuals carrying the wild‐type (GG). The rs1065852 polymorphism significantly increased the risk of LC development in all three genetic models (Dominant, AA/ AG vs GG, adjusted OR = 1.36, 95% CI = 1.03‐1.80, *P* = 0.030; Recessive, AA vs GG/AG, adjusted OR = 1.64, 95% CI = 1.22‐2.20, *P* = 0.001; Addictive, GG vs AA, adjusted OR = 1.34, 95% CI = 1.13‐1.60, *P* = 0.001, Table 5), with power values of 0.868, 0.997 and 0.879 respectively. We also found that the AG genotype of *CYP24A1* rs2762934 was associated with decreased risk of LC development (adjusted OR = 0.71, 95% CI = 0.51‐1.00, *P = *0.048). No statistically significant difference in the haplotype distributions between the case group and control group was observed for *CYP24A1 *(*P* > 0.05, Figure S1, Table S1).

**Table 4:** Candidate SNPs genotypes and the risk of lung cancer

Genotype	Case %		Control %		*P*	Crude OR (95%CI) *P*		Adjusted OR (95%CI) *P* ^a^		Stud power
rs2043449										
C/C	6	1.18	3	0.6	0.378	2.04 (0.51‐8.20)	0.317	2.08 (0.52‐8.39)	0.304	
C/T	74	15.5	63	12.5		1.20 (0.83‐1.72)	0.331	1.19 (0.83‐1.71)	0.347	
T/T	430	84.3	438	86.9		1.00 [Ref]		1.00 [Ref]		
**rs2762934**										
A/A	9	1.8	6	1.2	0.105	1.41 (0.50‐3.99)	0.519	1.42 (0.50‐4.03)	0.512	
A/G	73	14.3	96	19.0		**0.71 (0.51‐1.00)**	0.048^*^	**0.71 (0.51‐1.00)**	0.048^*^	**0.841**
G/G	428	83.9	402	79.8		1.00 [Ref]		1.00 [Ref]		
**rs1570669**										
A/A	83	16.1	71	14.1	0.428	1.11 (0.76‐1.61)	0.589	1.10 (0.75‐1.60)	0.635	
A/G	232	45.6	248	49.2		0.89 (0.68‐1.16)	0.385	0.88 (0.67‐1.15)	0.350	
G/G	195	38.3	185	36.7		1.00 [Ref]		1.00 [Ref]		
**rs6068816**										
T/T	56	11.1	52	10.4	0.455	1.17 (0.77‐1.79)	0.463	1.18 (0.77‐1.80)	0.443	
T/C	245	48.4	227	45.2		1.17 (0.90‐1.53)	0.230	1.17 (0.90‐1.52)	0.235	
C/C	205	40.5	223	44.4		1.00 [Ref]		1.00 [Ref]		
**rs2296241**										
A/A	96	18.9	96	19.1	0.981	0.97 (0.68‐1.39)	0.862	0.97 (0.68‐1.39)	0.873	
A/G	253	49.7	251	50.0		0.98 (0.74‐1.29)	0.868	0.98 (0.74‐1.30)	0.873	
G/G	160	31.4	155	30.9		1.00 [Ref]		1.00 [Ref]		
**rs1065852**										
A/A	144	28.2	97	19.5	0.003^*^	**1.80 (1.27‐2.56)**	0.001^*^	**1.83 (1.29‐ 2.59)**	0.001^*^	**0.998**
A/G	240	47.1	248	49.8		1.18 (0.88‐1.58)	0.284	1.18 (0.88‐ 1.59)	0.270	
G/G	126	24.7	153	30.7		1.00 [Ref]		1.00 [Ref]		

Notes

^*^ *P *≤ 0.05 value indicates statistical significance; *P* ^a^ adjusted for age and sex.

Bold indicates the siginificant value *P* ≤ 0.05 and the value of study power>0.8

Abbreviations: CI, confidence interval; ORs, odds ratios; Ref, reference category; SNP, single nucleotide polymorphism.

**Table 5:** Analysis of association between candidate SNPs and the risk of lung cancer in genetic model

Variable	Dominate model			Recessive model			Addictive model		
				Adjusted OR^a^ (95% CI) *P* ^a^ Study Power					
**rs2043449**	1.23 (0.86‐1.75)	0.250		2.03 (0.50‐8.19)	0.319		1.24 (0.90‐1.71)	0.194	
**rs2762934**	0.76 (0.55‐1.04)	0.087		1.50 (0.53‐4.26)	0.446		0.83 (0.62‐1.10)	0.190	
**rs1570669**	0.93 (0.72‐1.20)	0.561		1.18 (0.83‐1.66)	0.352		1.01 (0.84‐1.20)	0.938	
**rs6068816**	1.17 (0.91‐1.51)	0.210		1.09 (0.73‐1.62)	0.686		1.12 (0.92‐1.35)	0.257	
**rs2296241**	0.98 (0.75‐1.27)	0.856		0.99 (0.72‐1.35)	0.925		0.98 (0.82‐1.18)	0.862	
**rs1065852**	**1.36 (1.03‐1.80)**	0.030^*^	**0.868**	**1.64 (1.22‐2.20)**	0.001^*^	**0.997**	**1.34 (1.13‐1.60)**	0.001^*^	**0.879**

Notes

^*^P ≤ 0.05 value indicates statistical significance; *P* ^a^ adjusted for age and sex/

Bold indicates the siginificant value *P* ≤ 0.05 and the value of study power>0.8

Abbreviations: CI, confidence interval; ORs, odds ratios; SNP, single nucleotide polymorphism.

### 3.4 Linkage between candidate gene polymorphisms and LC in stratification analysis

As shown in Table 6, the AA genotype of *CYP2D6* rs1065852 was a risk factor for LC development in some subgroups (Males: adjusted OR = 1.60, 95% CI = 1.08‐2.39, *P* = 0.020; III‐IV stage: adjusted OR = 1.96, 95% CI = 1.27‐3.03, *P* = 0.002; Positive lymph node: adjusted OR = 2.08, 95% CI = 1.29‐3.33, *P* = 0.003; Adenocarcinoma[AC]: adjusted OR = 2.18, 95% CI = 1.34‐3.55, *P* = 0.002; Squamous cell carcinoma[SCC]: adjusted OR = 1.80, 95% CI = 1.10‐2.95, *P* = 0.020), whereas it was considered as a protective factor in females (Female: adjusted OR = 0.33, 95% CI = 0.16‐0.70, *P* = 0.004), with all power values more than 0.8 except in the addictive model of female. In addition, similar results were also found in some genetic models of part of subgroups (Table 6).

**Table 6:** Stratified analyses between *CYP2D6* rs1065852 polymorphism and lung cancer susceptibility

Variable	*CYP2D6* rs1065852 Adjusted OR (95%CI) *P^a^* Study Power										
	AA		AG		GG	(AA/AG) vs GG		AA vs (AG/GG)		AA vs GG	
Sex											
Male	1.60 (1.08‐2.39) 0.020^*^	**0.996**	0.85 (0.62‐1.18) 0.339		1.00	1.20 (0.88‐1.63) 0.263		1.57 (1.11‐2.21) 0.010^*^	**0.993**	1.25 (1.03‐1.53) 0.027^*^	**0.724**
Female	0.33 (0.16‐0.70) 0.004^*^	**0.999**	0.65 (0.35‐1.20) 0.168		1.00	0. 53 (0.30‐0.96) 0.035^*^	**0.986**	0.44 (0.24‐0.83) 0.011^*^	**0.999**	0.58 (0.40‐0.84) 0.004^*^	**0.999**
TNM Stage											
I‐II	1.47 (0.87‐2.47) 0.150		0.84 (0.53‐1.32) 0.445		1.00	1.01 (0.66‐1.54) 0.956		1.63 (1.04‐2.55) 0.032^*^	**0.998**	1.19 (0.91‐1.56) 0.206	
III‐IV	1.96 (1.27‐3.03) 0.002^*^	**0.999**	1.34 (0.92‐1.95) 0.129		1.00	1.51 (1.06‐2.16) 0.022^*^	**0.989**	1.62 (1.14‐2.32) 0.007^*^	**0.997**	1.40 (1.13‐1.74) 0.002^*^	**0.972**
Lymph node status											
Positive	2.08 (1.29‐3.33) 0.003^*^	**0.999**	1.38 (0.91‐2.08) 0.131		1.00	1.57 (1.07‐2.33) 0.023^*^	**0.999**	1.69 (1.15‐2.47) 0.008^*^	**0.999**	1.44 (1.14‐1.83) 0.003^*^	**0.988**
Negative	1.12 (0.65‐1.94) 0.682		0.81 (0.51‐1.29) 0.378		1.00	0.90 (0.59‐1.38) 0.627		1.27 (0.78‐2.05) 0.333		1.03 (0.78‐1.37) 0.818	
Type of cancer											
SCC	1.80 (1.10‐2.95) 0.020^*^	**0.977**	1.18 (0.77‐1.81) 0.441		1.00	1.35 (0.91‐2.02) 0.139		1.62 (1.07‐2.45) 0.022^*^	**0.997**	1.34 (1.04‐1.72) 0.023^*^	**0.921**
AC	2.18 (1.34‐3.55) 0.002^*^	**0.999**	1.02 (0.65‐1.59) 0.942		1.00	1.34 (0.89‐2.02) 0.163		2.15 (1.45‐3.21)1.16e‐4^*^	**0.999**	1.50 (1.16‐1.93) 0.002^*^	**0.997**
SCLC	1.28 (0.69‐2.38) 0.434		1.09 (0.65‐1.82) 0.744		1.00	1.14 (0.70‐1.86) 0.588		1.21 (0.72‐2.05) 0.471		1.13 (0.83‐1.54) 0.444	

Notes

^*^ *P* ≤ 0.05 value indicates statistical significance; *P* ^a^ adjusted for age and sex.

Bold indicates the siginificant value *P* ≤ 0.05 and the value of study power>0.8

Abbreviations: AC, adenocarcinoma; CI, confidence interval; ORs, odds ratios; SCC, Squamous cell carcinoma; SCLC, small cell lung cancer; TNM, tumor‐lymph node‐metastasis.

As shown in Table 7, the stratified analyses showed that the AG genotype of *CYP24A1* rs2762934 was associated with decreased LC risk in males (adjusted OR = 0.68; 95%CI = 0.46‐0.99, *P* = 0.046). A similar result was observed in recessive model of males (adjusted OR = 0.68, 95%CI = 0.47‐0.99, *P* = 0.044). We also identified that TC genotype of *CYP24A1* rs6068816 has potential effect on reducing the susceptibility to SCLC (SCLC, adjusted OR = 0.58, 95%CI = 0.36‐0.94, *P* = 0.026,Table 8).

**Table 7:** Stratified analyses between *CYP24A1* rs2762934 polymorphism and lung cancer susceptibility

Variable	***CYP24A1* rs2762934** Adjusted OR (95%CI) *P* ^a^ Study Power									
	AA		AG		GG (AA/AG) vs GG			AA vs (AG/GG)		AA vs GG
Sex										
Male	0.77 (0.20‐2.89) 0.693		0.68 (0.46‐0.99) 0.046^*^	**0.919**	1.00	0.68 (0.47‐0.99) 0.044^*^	**0.932**	0.82 (0.22‐3.08) 0.766		0.72 (0.51‐1.01) 0.057
Female	4.73 (0.54‐41.3) 0.160		0.84 (0.42‐1.68) 0.630		1.00	1.02 (0.54‐1.96) 0.942		4.86 (0.56‐42.33) 0.153		1.18 (0.68‐2.03) 0.560
TNM Stage										
I‐II	0.58 (0.07‐4.90) 0.615		0.72 (0.42‐1.23) 0.228		1.00	0.71 (0.42‐1.20) 0.202		0.61 (0.07‐5.18) 0.651		0.73 (0.44‐1.19) 0.200
III‐IV	1.29 (0.36‐4.62) 0.698		0.68 (0.44‐1.04) 0.074		1.00	0.71 (0.47‐1.07) 0.106		1.37 (0.38‐4.92) 0.626		0.78 (0.54‐1.13) 0.184
Lymph node status										
Positive	1.23 (0.30‐4.98) 0.775		0.69 (0.43‐1.10) 0.117		1.00	0.72 (0.46‐1.13) 0.151		1.31 (0.32‐5.29) 0.709		0.78 (0.52‐1.17) 0.229
Negative	0.59 (0.07‐5.01) 0.626		0.70 (0.40‐1.22) 0.208		1.00	0.69 (0.40‐1.19) 0.185		0.62 (0.07‐5.31) 0.665		0.71 (0.43‐1.18) 0.187
Type of cancer										
SCC	0.95 (0.18‐4.88) 0.947		0.73 (0.45‐1.18) 0.200		1.00	0.74 (0.46‐1.19) 0.211		1.00 (0.19‐5.14) 0.999		0.78 (0.50‐1.20) 0.253
AC	1.88 (0.51‐6.86) 0.342		0.74 (0.45‐1.21) 0.230		1.00	0.81 (0.50‐1.29) 0.375		1.97 (0.54‐7.21) 0.304		0.90 (0.59‐1.36) 0.619
SCLC	0.83 (0.10‐7.02) 0.863		0.54 (0.28‐1.06) 0.073		1.00	0.56 (0.29‐1.06) 0.077		0.91 (0.11‐7.70) 0.930		0.61 (0.33‐1.10) 0.100

Notes

^*^ *P* ≤ 0.05 value indicates statistical significance; *P* ^a^ adjusted for age and sex.

Bold indicates the siginificant value *P* ≤ 0.05 and the value of study power>0.8

Abbreviations: AC, adenocarcinoma; CI, confidence interval; ORs, odds ratios; SCC, Squamous cell carcinoma; SCLC, small cell lung cancer; TNM, tumor‐lymph node‐metastasis.

**Table 8:** Stratified analyses between *CYP24A1* rs6068816 polymorphism and lung cancer susceptibility

Variable	***CYP24A1* rs6068816** Adjusted OR (95%CI) *P* ^a^ Study Power									
	TT		TC		CC	AA + AG vs GG		AA vs AG + GG		AA vs GG	
Sex											
Male	1.32 (0.80‐2.17) 0.284		1.12 (0.83‐1.51) 0.465		1.00	1.15 (0.86‐1.53) 0.340		1.24 (0.77‐2.00) 0.376		1.14 (0.91‐1.42) 0.255	
Female	0.91 (0.40‐2.05) 0.811		1.36 (0.80‐2.33) 0.259		1.00	1.25 (0.75‐2.08) 0.391		0.77 (0.36‐1.66) 0.506		1.06 (0.73‐1.54) 0.762	
TNM Stage											
I‐II	0.98 (0.48‐2.02) 0.960		1.28 (0.85‐1.94) 0.235		1.00	1.23 (0.83‐1.83) 0.309		0.86 (0.43‐1.71) 0.665		1.09 (0.81‐1.48) 0.560	
III‐IV	1.21 (0.72‐2.02) 0.481		1.17 (0.84‐1.62) 0.347		1.00	1.18 (0.86‐1.60) 0.308		1.11 (0.68‐1.81) 0.676		1.12 (0.89‐1.41) 0.335	
Lymph node status											
Positive	0.82 (0.44‐1.52) 0.526		1.06 (0.75‐1.50) 0.740		1.00	1.02 (0.73‐1.42) 0.924		0.79 (0.44‐1.43) 0.445		0.96 (0.75‐1.25) 0.783	
Negative	1.49 (0.77‐2.90) 0.237		1.29 (0.84‐1.99) 0.249		1.00	1.33 (0.88‐2.01) 0.180		1.30 (0.70‐2.42) 0.402		1.24 (0.92‐1.68) 0.161	
Type of cancer											
SCC	1.29 (0.69‐2.42) 0.420		1.43 (0.98‐2.10) 0.063		1.00	1.41 (0.98‐2.03) 0.066		1.06 (0.59‐1.91) 0.845		1.23 (0.94‐1.62) 0.135	
AC	1.19 (0.64‐2.23) 0.586		1.45 (0.98‐2.13) 0.061		1.00	1.40 (0.96‐2.02) 0.078		0.97 (0.54‐1.75) 0.929		1.20 (0.91‐1.57) 0.198	
SCLC	0.94 (0.47‐1.90) 0.869		0.58 (0.36‐0.94)0.026^*^	**0.999**	1.00	0.65 (0.42‐1.01) 0.053		1.20 (0.61‐2.35) 0.596		0.81 (0.58‐1.14) 0.229	

Notes

^*^P ≤ 0.05 value indicates statistical significance; *P* ^a^ adjusted for age and sex.

Bold indicates the siginificant value *P* ≤ 0.05 and the value of study power>0.8

Abbreviations: AC, adenocarcinoma; CI, confidence interval; ORs, odds ratios; SCC, Squamous cell carcinoma; SCLC, small cell lung cancer; TNM, tumor‐lymph node‐metastasis.

*CYP20A1* rs2043449 polymorphism was found to increase the LC susceptibility in some subgroups (Male, CC/CT vs TT: adjusted OR = 1.50, 95% CI = 1.00‐2.24, *P* = 0.049 and CC vs TT: adjusted OR = 1.50, 95% CI = 1.03‐2.16, *P* = 0.033; III‐IV stage, CC vs TT: adjusted OR = 1.47, 95% CI = 1.01‐2.15, *P* = 0.044; SCLC, CC: adjusted OR = 5.36, 95% CI = 1.06‐27.21, *P* = 0.043 and CC vs CT/TT: adjusted OR = 5.27, 95% CI = 1.04‐26.66, *P* = 0.045, Table 9).

**Table 9:** Stratified analyses between CYP20A1 rs2043449 polymorphism and lung cancer susceptibility

Variable	***CYP20A1* rs2043449** Adjusted OR (95%CI) *P* ^a^ Study Power									
	CC		CT	TT	CC/CT vs TT		CC vs CT/TT		CC vs TT	
Sex										
Male	3.19 (0.64‐15.94) 0.158		1.42 (0.94‐2.16) 0.095	1.00	1.50 (1.00‐2.24) 0.049^*^	**0.916**	3.04 (0.61‐15.17) 0.176		1.50 (1.03‐2.16) 0.033^*^	0.123
Female	6.33e‐10 (0.00‐inf) 0.999		0.65 (0.30‐1.40) 0.270	1.00	0. 62 (0.29‐1.32) 0.211		0.00 (0.00‐inf) 0.999		0.60 (0.29‐1.25) 0.174	
TNM Stage										
I‐II	2.09e‐9 (0.00‐inf) 0.999		0.95 (0.53‐1.72) 0.872	1.00	0.91 (0.50‐1.63) 0.743		2.11e‐9 (0.00‐inf) 0.999		0.87 (0.49‐1.52) 0.620	
III‐IV	2.93 (0.65‐13.25) 0.162		1.41 (0.92‐2.15) 0.119	1.00	1.47 (0.97‐2.23) 0.067		2.79 (0.62‐12.60) 0.182		1.47 (1.01‐2.15) 0.044^*^	0.127
Lymph node status										
Positive	1.85 (0.31‐11.2) 0.504		1.46 (0.92‐2.32) 0.106	1.00	1.50 (0.94‐2.32) 0.088		1.75 (0.29‐10.57) 0.544		1.44 (0.95‐2.18) 0.086	
Negative	2.91 (0.46‐18.22) 0.255		0.75 (0.39‐1.45) 0.399	1.00	0.84 (0.45‐1.57) 0.591		3.00 (0.48‐18.76) 0.241		0.95 (0.54‐1.65) 0.841	
Type of cancer										
SCC	0.81 (0.08‐8.40) 0.862		1.34 (0.82‐2.21) 0.245	1.00	1.32 (0.81‐2.15) 0.272		0.78 (0.08‐8.04) 0.834		1.26 (0.80‐1.99) 0.326	
AC	1.07 (0.11‐10.51) 0.957		1.00 (0.58‐1.72) 0.999	1.00	1.00 (0.59‐1.71) 0.992		1.07 (0.11‐10.50) 0.957		1.01 (0.61‐1.65) 0.983	
SCLC	5.36 (1.06‐27.21) 0.043^*^	**0.979**	1.14 (0.60‐2.18) 0.682	1.00	1.34 (0.74‐2.44) 0.335		5.27 (1.04‐26.66) 0.045^*^	**0.991**	1.46 (0.87‐2.45) 0.148	

Notes

^*^ *P* ≤ 0.05 value indicates statistical significance; *P* ^a^ adjusted for age and sex.

Bold indicates the siginificant value *P* ≤ 0.05 and the value of study power>0.8

Abbreviations: AC, adenocarcinoma; CI, confidence interval; ORs, odds ratios; SCC, Squamous cell carcinoma; SCLC, small cell lung cancer; TNM, tumor‐lymph node‐metastasis.

Power calculations confirm that the sample size was large enough to discover the differences among cases and controls in candidate SNPs because the power values were more than 0.8 except in some genetic models in stratified analysis (Table 3, 4, 5, 6, 7, 8). However, no significant association was observed between other SNPs and LC in stratification analysis (Tables S2 and S3).

### 3.5 The expression and prognostic value of candidate genes in LC patients

**Figure 1:**
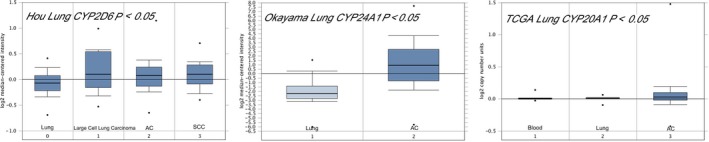
Expression of candidate genes in lung cancer; Abbreviations: AC, adenocarcinoma; SCC, Squamous cell carcinoma

**Figure 2:**
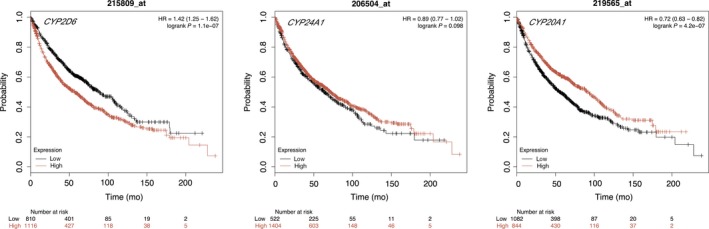
The prognostic value of gene level in lung cancer patients; 215809_at = CYP2D6, 206504_at = CYP24A1, 219565_at = CYP20A1, Hazard Rate = HR, *P* ≤ 0.05 value indicates statistical significance

As shown in Figure 1, we found the expressions of *CYP2D6* were significantly up‐regulated in large cell lung carcinoma, AC and SCC patients compared with the normal samples (*P* < 0.05), and *CYP24A1* and *CYP20A1* were found overexpressed in the AC (*P* < 0.05). Kaplan‐Meier curve and log‐rank test analyses revealed that the increased *CYP2D6* level and decreased *CYP20A1* level were significantly associated with poor overall survival (OS) in all LC patients (HR = 1.42, 95%CI = 1.25‐1.62, *P* = 1.1e‐07; HR = 0.72, 95%CI = 0.63‐0.82, *P* = 4.2e‐7 respectively, Figure 2). There was no significant association between expression of *CYP24A1* and OS of LC (*P* = 0.098).

## 4 DISCUSSION

Based on the metabolic characteristics of CYPs, we hypothesized that their polymorphisms were related to the risk of LC development. This study validated the potential relationship between four SNPs in three CYPs and risk of LC development. We found that rs2762934 and rs6068816 in *CYP24A1* decreased the risk of LC development in males and SCLC respectively, and *CYP20A1* rs2043449 was identified as a risk factor of LC development in males, III‐IV stage, and SCLC subgroups. The most significant discovery is that the “A” allele and “AA” genotype of *CYP2D6* rs1065852 confer risk to LC, especially in the cases of III‐IV stage AC, SCC, positive lymph node and males. These results made us assume that the susceptibility to LC may in part be defined by the individual's genetic background of CYPs.

*CYP24A1* encodes 24‐hydroxylase, the rate‐limiting enzyme that catalyzes the inactivation of 1,25(OH)2D3 (1,25‐D3), which is considered as a proto‐oncogene.^27^ High 1,25‐D3 levels have antidifferentiation and antiproliferation activities in human LC cell lines.^28^ Earlier researches reported that the gene copy number of *CYP24A1* is aberrantly amplified in several cancers,^29^, ^30^ and spontaneous upregulation of *CYP24A1* is a negative prognosticator of survival in lung, breast, ovarian and colon cancer.^31^, ^32^, ^33^ *CYP24A1* rs6068816 might promote the progression of colon cancer.^34^ Wu et al found that mutated homozygous *CYP24A1* rs6068816 was significantly related to the decrease of the risk of non‐small cell lung cancer (NSCLC) development among Chinese people.^35^ Liu et al found *CYP24A1* rs2762934 contributed to the risk of food hypersensitivity and breast cancer.^36^, ^37^ In the present study, we found that rs2762934 and rs6068816 in *CYP24A1* are protective factors to LC for males and in SCLC respectively. Furthermore, *CYP24A1* was significantly upregulated in LC. Nithya Ramnath et al revealed that promoter DNA hypermethylation of *CYP24A1* is a key mechanism regulating *CYP24A1* expression in LC. 38 *CYP24A1* has a promoter region that is rich in CpG islands, and transcriptional silencing of the *CYP24A1* gene is caused by promoter hypermethylation that would be conductive to 1,25‐D3 antiproliferative effects in LC. Because the amino acid sequence of *CYP24A1* is not affected by rs6068816 due to synonymous polymorphisms, the SNPs, located in silencers or enhancers of splicing regions, have an effect on the phenotype of biologic activities by influencing the efficiency of mRNA splicing. The rs2762934 plays a crucial role in intron and 3′UTR variant. RNA‐binding proteins combined with cis‐acting elements in the 3'UTR region to regulate protein synthesis by influencing mRNA abundance.^39^ Both the variation of 3'UTR sequence and abnormal expression of trans‐acting factors can significantly influence the transcription and expression of target genes. A possible reason for the association of rs6068816 and rs2762934 in *CYP24A1* with decreased risk of SCLC and LC development in males is the alternation of posttranscription process and dysfunction of the proteins. For all we know, this has been the first clinic study to estimate the relationship between rs2762934 in *CYP24A1* and LC susceptibility.

*CYP2D6* is a member of the CYP450 superfamily of enzymes involved in the metabolism of therapeutic drugs and is a potential susceptibility factor for certain environmental agent‐induced diseases.^40^, ^41^, ^42^ It plays an important role particularly in the metabolism of PAH, nicotine and other carcinogens related to LC. To date, there have been studies that have shown that genetic polymorphisms of *CYP2D6* increase the susceptibility to numerous cancers. Studies have indicated that polymorphisms of *CYP2D6* imposed an increased risk of breast cancer and esophageal squamous cell carcinoma in those people with a family history of cancers.^43^, ^44^ Zienolddiny S et al found that *CYP2D6* and *CYP1B1* increased genetic susceptibility to NSCLC.^45^ In addition, Lee JY et al showed that hydroxychloroquine metabolism was related to *CYP2D6* rs1065852 polymorphisms.^46^

It has been confirmed that *CYP2D6* participates in the metabolism of the tobacco, nitrosamine, nicotine‐derived nitrosamine ketone, nicotine, cotinine, as well as the activation of nitrosamine, all of which are common carcinogenetic agents of LC.^47^, ^48^ In China, the proportion of smoking and tobacco‐attributed mortality is much higher in males than in females.^49^ SCC is one of the most common pathological type of smoking‐related LC.^50^ Therefore, it could be assumed that the significant increased risk of LC in males and SCC patients by rs1065852 may be caused by the accumulation of smoking‐related genetic damage. Meanwhile, high levels of *CYP2D6* was found in SCC and AC, and survival analysis also confirmed the poor prognosis of LC caused by *CYP2D6*. *CYP2D6* rs1065852 is located in the intron region of *CYP2D6* gene and involved in intron mutation. Intron is important for functions in RNA stability, regulation of gene expression and alternative splicing. Misregulation of alternative splicing is known contribute to tumorigenesis,^51^ and the missense variant of base near the splice site could lead to protein and amino acid change due to aberrant splicing. It might be an assumption that the polymorphism of rs1065852 may be involved in the development of LC by influencing the biological function of gene products and mRNA splicing. As we know, no study has validated the association between *CYP2D6* rs1065852 and LC susceptibility, and the present study is the first of its kind to verify the correlation between *CYP2D6* rs1065852 and the increased of LC in Asians.

Stratified analysis also revealed significant associations between *CYP20A1* rs2043449 and increased risk of LC in males, III‐IV stage, and SCLC subgroups. Although high level of *CYP20A1* predicts a better prognosis in survival analysis, the conflict between these two outcomes might be due to the sample size, territory and racial differences. Previous studies showed that *CYP20A1* is expressed in the human hippocampus and substantia nigra, suggesting its involvement in brain and early development.^52^ As far as we know, *CYP20A1* was considered as “orphan” CYP with no functional information.^53^ Therefore, the mechanism of rs2043449 affecting tumor susceptibility in these subgroups remains unclear; further functional analysis of *CYP20A1* in these subgroups may help to clarify the relevant genetic effects of LC pathogenesis.

In this study, we identified four novel loci in three genes that show a significant linkage with LC development, and observed the expression of candidate genes in LC and the relationship between poor prognosis of LC and two genes. Although the results showed strong statistical significance, there are still several potential limitations in the present research. First, LC is a very heterogeneous disease with many other risk factors, and more genes need to be included in follow‐up studies. Second, the study is conducted among only in the Chinese Han people in Northwest China, further investigations are needed to confirm these associations in other populations. Third, the sample size was not large enough to support some genetic models in stratified analyses. Finally, the smoking data of the samples were not collected, and further study is needed to improve the deficiencies of this research.

## 5 CONCLUSION

In this study, we systematically evaluated the association of candidates genes and LC risk in a case‐control study including 510 cases and 504 healthy controls. And finally we found the significant relationship of *CYP2D6* rs1065852, *CYP20A1* rs2043449, *CYP24A1* rs2762934, and *CYP24A1* rs6068816 with susceptibility to LC. In addition, we explored the overexpression of candidate genes in LC and estimated the relationship between LC prognosis and genes expression level in survival analysis using Oncomine and Kaplan‐Meier Plotter database, which could potentially contribute to elucidate the etiology of LC and be used as diagnostic and prognostic molecular markers for LC in Northwest Chinese Han population.

## CONFLICT OF INTEREST

The authors made no disclosures.

## Data Availability

The data that support the findings of this study are available from the corresponding author upon reasonable request.
