# Supplementary figures and images for: Gene polymorphism of cytochrome P450 significantly affects lung cancer susceptibility
Source: Cancer Med. 2019 Jul 1;8(10):4892–905. doi: 10.1002/cam4.2367 (PMC6712450; doi:10.1002/cam4.2367)

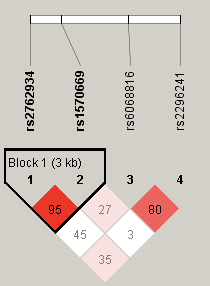

Supplement: Supplementary file 1 [file CAM4-8-4892-s001.tiff]
