# Supplementary material for: Gene polymorphism of cytochrome P450 significantly affects lung cancer susceptibility
Source: Cancer Med. 2019 Jul 1;8(10):4892–905. doi: 10.1002/cam4.2367 (PMC6712450; doi:10.1002/cam4.2367)
Supplement: Supplementary file 2 [file CAM4-8-4892-s002.docx]

**Supplementary tables**

**Supplementary Table 1** Haplotype frequencies and the association with lung cancer susceptibility

| Block ID | Chromosome | Gene | SNPs | Haplotype | Adjusted OR(95%CI) *p*^a^ | |
| --- | --- | --- | --- | --- | --- | --- |
| 1 | Chr 20 | CYP24A1 | rs2762934\| rs1570669 | AA | 0.91(0.75-1.10) | 0.330 |
|  |  |  |  | GA | 0.84(0.63-1.10) | 0.241 |
|  |  |  |  | GG | 1.09(0.90-1.32) | 0.379 |

**Notes:***^*^p*≤0.05value indicates statistical significance; *p*^a^ adjusted for age and sex

**Abbreviations:** SNP, single-nucleotide polymorphism; ORs, odds ratios; CI, confidence interval

**Supplementary Table 2** Stratified analyses between CYP24A1 rs1570669 polymorphism and lung cancer susceptibility

| Variable | CYP24A1 rs1570669  Adjusted OR(95%CI) *p^a^* | | | | | |
| --- | --- | --- | --- | --- | --- | --- |
|  | AA | AG | GG | (AA/AG) vs GG | AA vs (AG/GG) | AA vs GG |
| Sex |  |  |  |  |  |  |
| Male | 0.99(0.64-1.52) 0.956 | 0.99(0.73-1.35) 0.964 | 1.00 | 0.99(0.74-1.33) 0.955 | 0.99(0.67-1.48) 0.968 | 0.99(0.81-1.22) 0.952 |
| Female | 1.48(0.68-3.23) 0.327 | 0.60(0.35-1.05) 0.075 | 1.00 | 0.75(0.44-1.27) 0.279 | 1.98(0.97-4.03) 0.060 | 1.05(0.73-1.51) 0.799 |
| TNM Stage |  |  |  |  |  |  |
| I-II | 1.07(0.60-1.92) 0.819 | 0.89(0.58-1.37) 0.608 | 1.00 | 0.93(0.62-1.40) 0.741 | 1.14(0.67-1.95) 0.631 | 1.00(0.75-1.33) 0.986 |
| III-IV | 0.98(0.61-1.58) 0.932 | 1.02(0.73-1.42) 0.905 | 1.00 | 1.01(0.74-1.39) 0.945 | 0.97(0.62-1.50) 0.885 | 1.00(0.80-1.25) 0.980 |
| Lymph node status |  |  |  |  |  |  |
| Positive | 1.03(0.62-1.71) 0.923 | 0.92(0.64-1.33) 0.662 | 1.00 | 0.95(0.67-1.33) 0.749 | 1.07(0.67-1.72) 0.768 | 0.99(0.78-1.27) 0.940 |
| Negative | 1.12(0.60-2.06) 0.726 | 1.03(0.66-1.61) 0.898 | 1.00 | 1.05(0.69-1.60) 0.823 | 1.10(0.63-1.91) 0.743 | 1.05(0.78-1.41) 0.742 |
| Type of cancer |  |  |  |  |  |  |
| SCC | 1.10(0.65-1.86) 0.728 | 0.88(0.60-1.30) 0.520 | 1.00 | 0.93(0.65-1.34) 0.698 | 1.18(0.73-1.91) 0.505 | 1.01(0.78-1.31) 0.941 |
| AC | 1.29(0.76-2.20) 0.339 | 0.91(0.61-1.35) 0.637 | 1.00 | 0.99(0.68-1.44) 0.971 | 1.37(0.85-2.21) 0.202 | 1.09(0.84-1.42) 0.520 |
| SCLC | 1.05(0.54-2.04) 0.877 | 0.89(0.55-1.43) 0.619 | 1.00 | 0.92(0.59-1.45) 0.728 | 1.13(0.61-2.07) 0.701 | 0.99(0.72-1.37) 0.961 |

**Notes:** *^*^p*≤0.05value indicates statistical significance; *p*^a^ adjusted for age and sex

**Abbreviations:** ORs, odds ratios; CI, confidence interval; TNM, tumor-lymph node-metastasis; SCC, Squamous cell carcinoma; AC, adenocarcinoma; SCLC, small cell lung cancer

**Supplementary Table 3** Stratified analyses between CYP24A1 rs2296241 polymorphism and lung cancer susceptibility

| Variable | ***CYP24A1* rs2296241**  Adjusted OR(95%CI) *p^a^* | | | | | |
| --- | --- | --- | --- | --- | --- | --- |
|  | AA | AG | GG | (AA/AG) vs GG | AA vs (AG/GG) | AA vs GG |
| Sex |  |  |  |  |  |  |
| Male | 1.00(0.66-1.53) 0.993 | 0.85(0.62-1.18) 0.339 | 1.00 | 0.89(0.65-1.21) 0.460 | 1.11(0.77-1.60) 0.588 | 0.98(0.80-1.21) 0.850 |
| Female | 0.90(0.45-1.81) 0.764 | 1.51(0.85-2.67) 0.161 | 1.00 | 1.28(0.75-2.17) 0.365 | 0.71(0.38-1.32) 0.278 | 1.00(0.71-1.40) 0.984 |
| TNM Stage |  |  |  |  |  |  |
| I-II | 0.75(0.43-1.31) 0.314 | 0.68(0.44-1.06) 0.086 | 1.00 | 0.70(0.47-1.05) 0.087 | 0.93(0.56-1.55) 0.787 | 0.83(0.63-1.10) 0.196 |
| III-IV | 1.06(0.67-1.67) 0.814 | 1.27(0.89-1.81) 0.191 | 1.00 | 1.21(0.86-1.70) 0.274 | 0.91(0.61-1.35) 0.631 | 1.05(0.84-1.31) 0.649 |
| Lymph node status |  |  |  |  |  |  |
| Positive | 1.08(0.65-1.77) 0.773 | 1.19(0.80-1.75) 0.388 | 1.00 | 1.16(0.80-1.67) 0.442 | 0.97(0.63-1.48) 0.872 | 1.05(0.83-1.34) 0.675 |
| Negative | 0.69(0.38-1.24) 0.213 | 0.69(0.57-1.08) 0.102 | 1.00 | 0.69(0.46-1.05) 0.080 | 0.85(0.50-1.45) 0.556 | 0.80(0.60-1.07) 0.135 |
| Type of cancer |  |  |  |  |  |  |
| SCC | 0.96(0.58-1.59) 0.865 | 0.85(0.58-1.27) 0.422 | 1.00 | 0.88(0.60-1.28) 0.499 | 1.06(0.68-1.65) 0.809 | 0.96(0.75-1.24) 0.755 |
| AC | 0.64(0.37-1.11) 0.116 | 0.90(0.60-1.34) 0.595 | 1.00 | 0.82(0.56-1.20) 0.315 | 0.69(0.42-1.13) 0.138 | 0.82(0.63-1.06) 0.135 |
| SCLC | 1.35(0.69-2.64) 0.389 | 1.58(0.93-2.70) 0.091 | 1.00 | 1.52(0.91-2.54) 0.111 | 0.99(0.57-1.73) 0.970 | 1.18(0.86-1.63) 0.298 |
| SCLC | 1.35(0.69-2.64) 0.389 | 1.58(0.93-2.70) 0.091 | 1.00 | 1.52(0.91-2.54) 0.111 | 0.99(0.57-1.73) 0.970 | 1.18(0.86-1.63) 0.298 |

**Notes:** *^*^p*≤0.05value indicates statistical significance; *p*^a^ adjusted for age and sex

**Abbreviations:** ORs, odds ratios; CI, confidence interval; TNM, tumor-lymph node-metastasis; SCC, Squamous cell carcinoma; AC, adenocarcinoma; SCLC, small cell lung cancer
